# BRD4 bimodal binding at promoters and drug-induced displacement at Pol II pause sites associates with I-BET sensitivity

**DOI:** 10.1186/s13072-019-0286-5

**Published:** 2019-07-02

**Authors:** P. Khoueiry, A. Ward Gahlawat, M. Petretich, A. M. Michon, D. Simola, E. Lam, E. E. Furlong, V. Benes, M. A. Dawson, R. K. Prinjha, G. Drewes, P. Grandi

**Affiliations:** 1 Cellzome GmbH, a GSK Company, Heidelberg, Germany; 2 Department of Biochemistry and Molecular Genetics, Faculty of Medicine, American University of Beirut, Beirut, Lebanon; 3 Peter MacCallum Cancer Center, Melbourne, Australia; 4 European Molecular Biology Laboratory (EMBL), Genome Biology Unit, Heidelberg, Germany; 5 European Molecular Biology Laboratory (EMBL), Genomics Core Facility, Heidelberg, Germany; 6 Epigenetics DPU, GSK Medicines Research Centre, Stevenage, UK; 7 Target Science Computational Biology, GSK Medicines Research Centre, Upper Providence, USA

**Keywords:** Bromodomain proteins, Sensitivity and resistance to drug treatment, Regulatory regions, Promoters, TSS, Leukemia

## Abstract

**Background:**

Deregulated transcription is a major driver of diseases such as cancer. Bromodomain and extra-terminal (BET) proteins (BRD2, BRD3, BRD4 and BRDT) are chromatin readers essential for maintaining proper gene transcription by specifically binding acetylated lysine residues. Targeted displacement of BET proteins from chromatin, using BET inhibitors (I-BETs), is a promising therapy, especially for acute myeloid leukemia (AML), and evaluation of resistance mechanisms is necessary to optimize the clinical efficacy of these drugs.

**Results:**

To uncover mechanisms of intrinsic I-BET resistance, we quantified chromatin binding and displacement for BRD2, BRD3 and BRD4 after dose response treatment with I-BET151, in sensitive and resistant in vitro models of leukemia, and mapped BET proteins/I-BET interactions genome wide using antibody- and compound-affinity capture methods followed by deep sequencing. The genome-wide map of BET proteins sensitivity to I-BET revealed a bimodal pattern of binding flanking transcription start sites (TSSs), in which drug-mediated displacement from chromatin primarily affects BRD4 downstream of the TSS and prolongs the pausing of RNA Pol II. Correlation of BRD4 binding and drug-mediated displacement at RNA Pol II pause sites with gene expression revealed a differential behavior of sensitive and resistant tumor cells to I-BET and identified a BRD4 signature at promoters of sensitive coding and non-coding genes.

**Conclusions:**

We provide evidence that I-BET-induced shift of Pol II pausing at promoters via displacement of BRD4 is a determinant of intrinsic I-BET sensitivity. This finding may guide pharmacological treatment to enhance the clinical utility of such targeted therapies in AML and potentially other BET proteins-driven diseases.

**Electronic supplementary material:**

The online version of this article (10.1186/s13072-019-0286-5) contains supplementary material, which is available to authorized users.

## Background

Since the identification of the first epigenetic mechanism of oncogene regulation in 1983 [1], several studies have highlighted the effect of chromatin state alterations on cancer initiation and progression [2–4]. In addition to DNA hyper- and hypo-methylation, post-translational histone modifications by chromatin writers, erasers and readers are major epigenetic alterations that occur in cancer [5, 6]. Deeper insights into the biological function of histone modifications in cancer pathogenesis drove drug discovery approaches to target chromatin readers. As chromatin readers recognize modified histones, this orchestrates a binding cascade of chromatin effectors that ultimately control transcription [7].

Bromodomain (BD)-containing proteins, 61 in humans, are readers of acetyl-lysine residues present on histones and other proteins [8]. Members of this family include the bromodomain and extra-terminal repeat (BET) proteins composed of the four paralogs BRD2, BRD3, BRD4 and BRDT [9] with the latter only expressed in testes. BET proteins contain two conserved bromodomains, BD1 and BD2, which bind acetylated lysine residues in the histone tails of nucleosomes and of certain transcription factors (TFs), thereby forming a landing pad for transcriptional regulators on chromatin [10]. The occupancy of BET proteins is enriched on promoters of active genes, in addition to being frequently found on enhancers and super-enhancers (a large cluster of active enhancers) where they co-localize with the Med1 (Mediator) complex, RNA polymerase II (Pol II) and H3K27ac signals [11, 12]. BRD4 is the only member of the BET protein family which harbors a C-terminal domain that recruits the positive transcription elongation factor b (P-TEFb) to the TSS. P-TEFb, in turn, releases RNA Pol II from a paused state into an elongation state [13, 14], thereby enhancing transcription of oncogenes such as c-Myc and BCL2 [15–18]. Besides the role in transcription elongation at the TSS, BRD4 is also involved in *cis*-regulation of genes by binding at enhancers and facilitating eRNA transcription [11, 12, 19, 20].

The discovery of BRD3- and BRD4-NUT fusion genes in midline carcinoma [21] provided genetic evidence for a role of BET proteins in carcinogenesis. This finding led to the development of small molecule BET inhibitors (I-BET) such as I-BET151 and JQ1 which bind to the bromodomain pocket of BET proteins, thereby displacing or preventing recruitment of BRD2, BRD3 and BRD4 from or to chromatin [22–24]. In general, inhibition of BET proteins has shown efficacy for both hematological cancers including leukemia, lymphoma, multiple myeloma and for solid tumors such as NMC (Nut midline carcinoma), prostate cancer and ovarian cancer [10, 15, 25–29]. I-BETs have also shown potential use in the treatment of atherosclerosis and cardiovascular diseases [30–32]. Through its interaction and recruitment of P-TEFb at promoters of growth-associated genes, BRD4 inhibition interferes with transcription initiation and elongation [14, 33] causing profound effects on the cell cycle. For instance, JQ1 treatment of patient-derived NUT cancer cell lines induces terminal differentiation and has anti-proliferative effects in patient-derived xenograft models [17, 23, 25]. Additionally, JQ1-dependent in vivo inhibition of BET protein bromodomains in murine models of multiple myeloma showed a prolonged overall survival compared to vehicle control [16, 34].

I-BET-dependent displacement of BRD4 was observed at promoter as well as on enhancers and super-enhancers of target genes [35–37] such as the MYC oncogene and CCND2 [12, 16]. However, it remains unclear whether individual BET proteins bound at gene loci manifest a differential sensitivity to I-BET and whether there is a relationship between the extent of BRD2/BRD3/BRD4 displacement and the intrinsic response to the drug.

To understand the mechanism of intrinsic sensitivity and resistance to I-BET, we have undertaken an integrative epigenomic, chemo-genomic and transcriptomic approach by assessing the effects of increasing concentrations of I-BET151 on chromatin-bound BRD2, BRD3 and BRD4 and gene expression in one resistant (K562) and one sensitive (MV4;11) leukemia cell lines. Analysis of the spatial patterns of BRD2, BRD3 and BRD4 occupancy at promoters revealed a bimodal signature around the TSS and indicates that I-BET-mediated BRD4 displacement 150–180 bp downstream of the TSS is linked to strong and selective downregulation of gene expression in the I-BET-sensitive cell line. Finally, a handful of gene loci including four non-coding RNAs was identified for which BET protein binding is selectively affected upon I-BET treatment in MV4;11 compared to K562 cells as well as a transcriptional enrichment signature which hints at the molecular mechanism of intrinsic sensitivity to I-BET drugs.

## Results

### Genome-wide binding of BET proteins exhibits dose-dependent displacement upon I-BET treatment

**Fig. 1:**
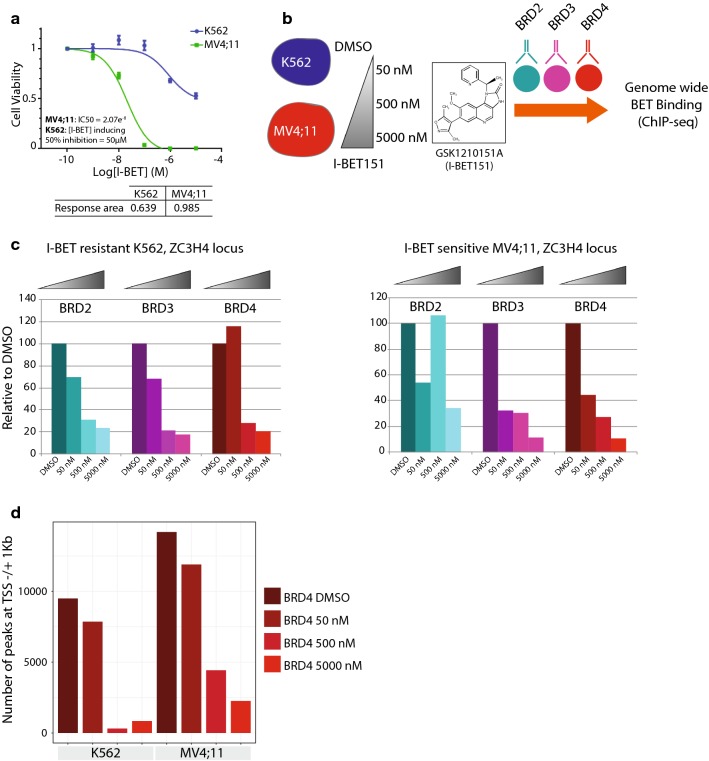
BET proteins displacement from chromatin is compound dose dependent. **a** Proliferation assays in sensitive (MV4;11) and resistant (K562) cells treated with increasing concentrations of I-BET151. Compound IC50 (M) or compound concentration which induces 50% inhibition of cell viability as well as the response area (see “Materials and methods” section for the calculation) is shown below the graph. **b** Experimental design followed in the study and I-BET151 structure. **c** Bar plot showing the ChIP-qPCR enrichment relative to DMSO for the ZC3H4 locus in K562 and MV4;11 for cells treated with three concentrations of I-BET151 and DMSO as a control. **d** Barplot showing the number of peaks called for BRD4 at TSS for cells treated with three concentrations of I-BET151 and DMSO as a control and for both cell lines

To evaluate I-BET resistance in leukemia, we used the pan-BET inhibitor GSK1210151A (I-BET151) [15], in two cell line models: the CML (chronic myeloid leukemia) I-BET-resistant K562 and the AML (acute myeloid leukemia) I-BET-sensitive MV4;11. Proliferation of K562 cells is driven by a BCR-ABL translocation, and these cells exhibit resistance to I-BET151 treatment up to micromolar concentrations [15]. On the contrary, MV4;11 cells are dependent on a MLL-AF4 fusion and are sensitive to I-BET151 anti-proliferative effect in the nanomolar range (Fig. 1a). To characterize the consequences of I-BET151 treatment on BET proteins bound to chromatin, we undertook a comparative approach by incubating MV4;11 and K562 cells with increasing concentrations of the inhibitor, followed by ChIP-seq for BRD2, BRD3 and BRD4 (Fig. 1b, “Materials and methods” section). For each cell line and condition (vehicle, I-BET151 50 nM, 500 nM or 5000 nM), two replicates exhibiting significant enrichment of known BET proteins-bound loci (verified by qPCR, “Materials and methods” section, Fig. 1c and Additional file 1: Table S1) were subjected to high-throughput sequencing.

Quantitative PCR on both cell lines showed general and gradual decrease in BET proteins binding upon treatment with increasing inhibitor concentration (Fig. 1c).

Peak calling based on a MACS2-derived pipeline [38] followed by joining operations was performed to call the final set of peaks and filter low-confidence ones (Additional file 2: Figure S1, “Materials and methods” section).

Interestingly, more BRD4 binding was observed in the I-BET-sensitive MV4;11 cells compared to the I-BET-resistant K562 cells (Fig. 1d, Additional file 3: Figure S2 and Additional file 4: Table S2). For K562, we observed a substantial reduction in peak counts for all three BET proteins at and above 500 nM of I-BET151 treatment. In contrast, a differential pattern was seen in MV4;11: BRD3 and BRD4 showed a clear decrease in peak numbers concomitant with increasing I-BET151 concentrations, while BRD2 showed an unusual binding scheme with more peaks observed at 500 nM, and generally less peaks compared to BRD3 and BRD4 (Additional file 3: Figure S2 and Additional file 4: Table S2) which could also be explained by the poor quality of the BRD2 antibody in ChIP-seq.

In general, the measured enrichment of BRD2, BRD3 and BRD4 peaks at promoters and H3K27ac marked regions (approximately 78% of all peaks, Additional file 3: Figure S2C and Additional file 4: Table S2), is in line with the role of BET proteins in cis and trans transcriptional regulation.

### BRD4 is the most sensitive BET paralog to I-BET treatment on enhancers and promoters

**Fig. 2:**
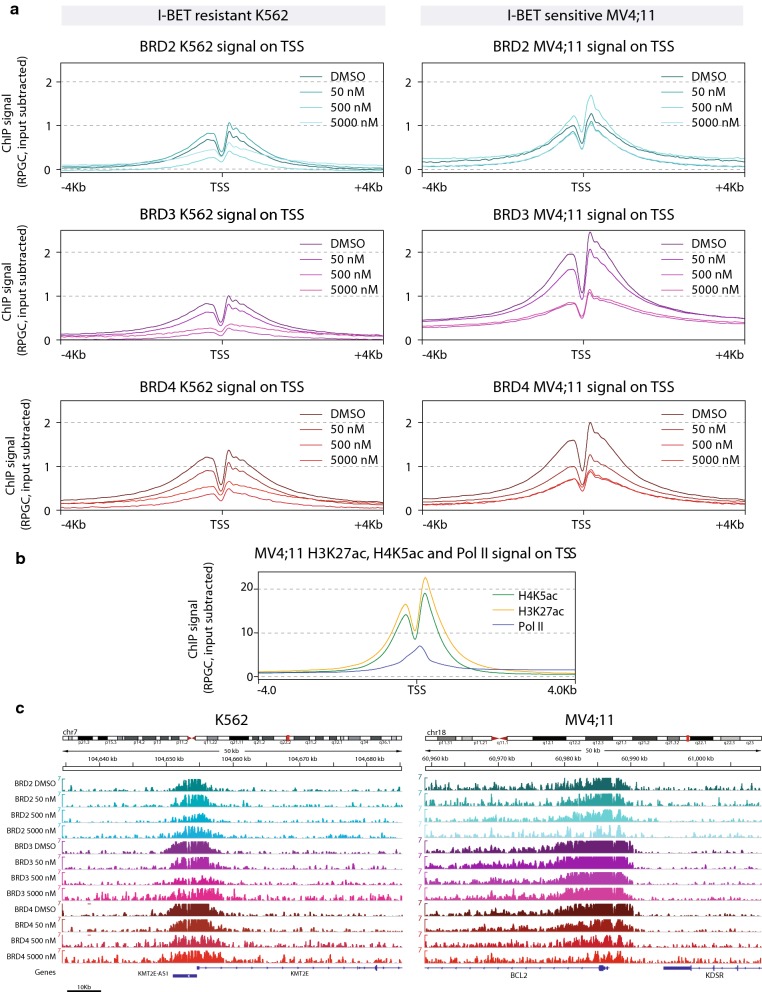
Genome-wide I-BET151 dose response of BET proteins binding profiles. **a** Genome-wide ChIP-seq profiles on TSS −/+ 4 Kb for BRD2, BRD3 and BRD4. All ChIP-seq profiles are RPGC (Reads Per Genomic Content) normalized followed by input subtraction. **b** ChIP-seq profiles obtained as in “a” for H3K27ac, H4K5ac and Pol II in MV4;11 from previously published data (52). **c** Genome browser visualization of two typical loci in both cell lines: KMT2E locus in K562 (left panel) and BCL2 locus in MV4;11 (right panel) showing the enrichment and the associated gradual decrease in ChIP-seq signal for all three BET proteins in DMSO and compound-treated samples. The same color codes are used for the genome browser and genome-wide profiles. The Y-scale is the same for all conditions and both cell lines for comparison purposes. Scale is indicated in the lower left corner

Previous studies showed that treatment with I-BET results in partial displacement of BRD2, BRD3 and BRD4 from their chromatin-bound loci [12, 36, 39–42]. The displacement is observed at the TSS or proximal to it, at extended promoters as well as at distant enhancers and super-enhancers [12]. However, little is known about the correlation between genome-wide displacement of the individual BET protein paralogs and I-BET concentration in resistant and sensitive cell lines. To address this point, we compared BET proteins ChIP-seq signals at the promoters in K562 and MV4;11 (Fig. 2, “Materials and methods” section).

The aggregation of BET proteins ChIP-seq signals around all TSSs in a meta-profile showed that binding of the BET proteins is globally stronger in the I-BET-sensitive MV4;11 compared to the I-BET-resistant K562 (Fig. 2a), also reflected by the higher number of peaks called in MV4;11 cells (Additional file 4: Table S2). All BET proteins showed a marked bimodal distribution with one strong signal peak approximately 150–180 bp downstream of the TSS and a less pronounced peak located at approximately 300 bp upstream of the TSS (Fig. 2a), consistent with established patterns of histone tail acetylation at positioned nucleosomes flanking the nucleosome-depleted region over gene promoters [43]. Notably, the difference in signal intensity between the downstream and the upstream peak was stronger in the sensitive MV4;11 cells than in the resistant K562 cells (Fig. 2a, c). The bimodal distribution of the BET proteins signal around the TSS resembles the enrichment patterns of H4K5ac and H3K27ac at these sites, while Pol II peaks mainly downstream of the TSS (Fig. 2b) [24, 39, 42]. Interestingly, among the three paralogs, BRD4 showed a stronger displacement at the lowest concentration of the inhibitor (50 nM) in MV4;11 reflected by a lower ChIP-seq signal. This suggests that BRD4 is the most I-BET151-sensitive BET paralog (Fig. 2a).

Moreover, since BRD4 recruits the P-TEFb complex to chromatin which in turn binds to Pol II, a co-localization of Pol II with BRD4 is expected. Although Pol II ChIP signal peaks mostly downstream of the TSS, a shoulder in the peak is noticeable upstream of the TSS (Fig. 2b). Consistent with the role of BET proteins in transcriptional elongation, the downstream peak showed a broader signal than the upstream peak in both cell lines for all BET proteins and particularly for BRD4 (Fig. 2a, c).

Similar binding profiles were observed at cell-type-specific intergenic enhancers comprising typical enhancers and super-enhancers (Additional file 5: Figure S3, “Materials and methods” section). BRD2 binding on enhancers was less pronounced than BRD3 and BRD4 for both cell lines with BRD4 exhibiting a more pronounced displacement at 50 nM compared to BRD3 in the sensitive cell line MV4;11 (Additional file 5: Figure S3).

### I-BET-mediated displacement of BRD4 around the TSS identifies a small set of hypersensitive genes and defines four clusters of differentially sensitive promoters

Although BET proteins displacement from chromatin after I-BET treatment has been well described [12, 39, 40], spatial resolution of displacement around the TSS has not been assessed in detail. Since BRD4 binding is particularly sensitive to I-BET in MV4;11 (Fig. 2a), we focused our analysis on this paralog.

**Fig. 3:**
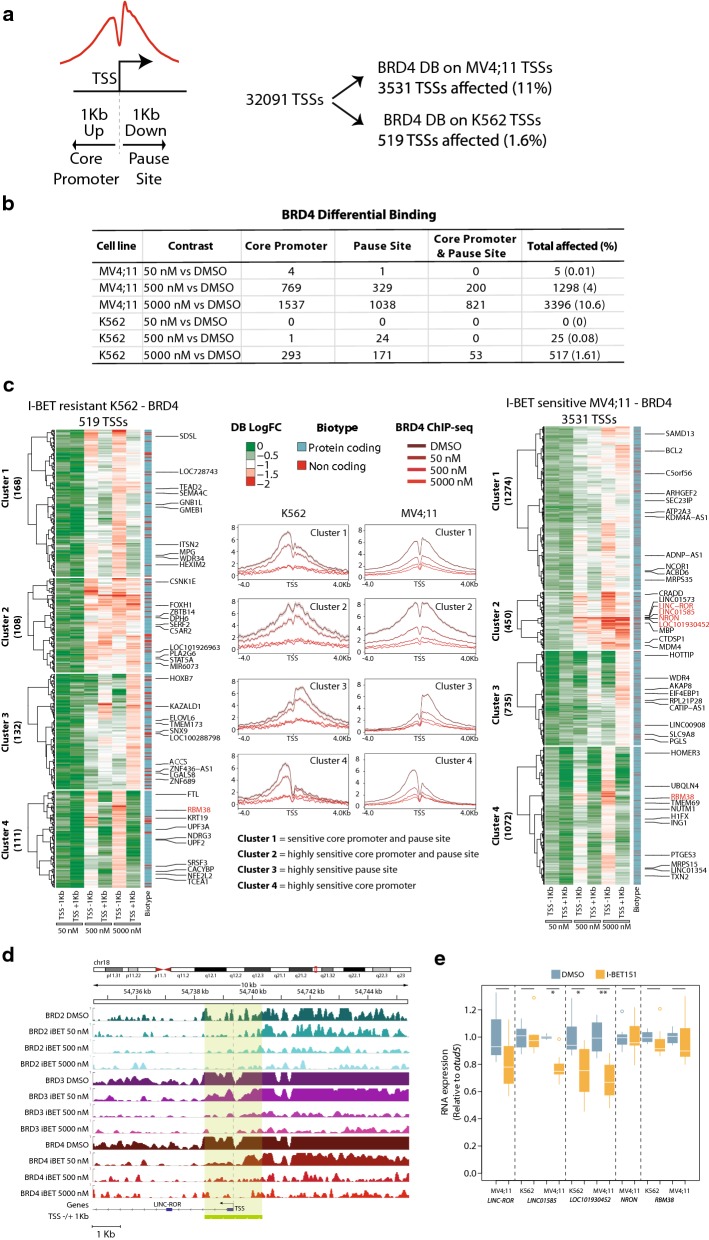
BRD4 displacement at TSSs correlates with cell sensitivity. **a** Representation of a TSS and the surrounding − 1 Kb (core promoter) and + 1 Kb (pause site) used for the DB analysis. Right panel shows the count of total TSSs and the number (and percentage) of TSSs affected (at − 1 Kb or + 1 Kb or both) in MV4;11 and K562. **b** Table containing TSS counts for significant cases of DB at core promoter and pause site or both. The “Total affected” column contains the number (and percentage) of TSSs affected at any of the two sites. **c** Hierarchical clustering of ChIP signal (log2 fold change or LFC) for TSSs (rows) with DB in − 1 Kb or + 1 Kb as defined above. For each of the three treatment conditions (50 nM, 500 nM or 5000 nM), LFC of signal at TSS − 1 Kb and TSS + 1 Kb (columns) in K562 (left, total of 519 TSSs with DB) and MV4;11 (right, total of 3531 TSSs with DB) are shown (“Materials and methods” section). In each cluster, a random set of genes is shown in addition to the 5 most sensitive genes in Cluster 2 showing differential binding at 50 nM I-BET in MV4;11 (in red) in addition to *BCL2* in Cluster 1. The category of each gene (protein coding or non-coding) is represented under the biotype column on the right to each heatmap. All four non-coding RNAs with a BRD4 DB at 50 nM I-BET151 in MV4;11 fall in cluster 2 for MV4;11 cells. For the K562 DB-based clustering (left panel heatmap), only the protein coding gene *RBM38* showed a significant decrease in binding. The middle panel represents metagene profiles of BRD4 ChIP-seq data for the corresponding clusters. Inferred names to the identified clusters are indicated below the metagene profiles. **d** Genome browser visualization of the *LINC*-*ROR* locus showing the strong decrease in ChIP signal for all BET proteins, and specifically BRD4, when treated with 50 nM I-BET151. Note the marked decrease at TSS + 1 Kb (pause site). The green line and gray shadowed box depict the TSS −/+ 1 Kb area. The scale is shown in the lower left corner. **e** RNA expression profiling by RT-qPCR on the five genes showing DB in MV4;11 cells at 50 nM of I-BET151. The expression value of each transcript is referred to the expression of a reference gene (OTUD5), and the mean of the DMSO for each comparison group is set to 1. Stars represent level of significance with (*) for *p* -value < 0.05, and (**) for *p* value < 0.005. Only cases with detectable expression are shown

We performed differential binding (DB) analysis using DESeq2 [44] (“Materials and methods” section) for BRD4 signal at − 1 Kb from the TSS (defined as core promoter) and at + 1 Kb from the TSS (defined as Pol II pause site) of the 32,091 annotated TSSs in the human genome (Fig. 3a).

The number of affected TSSs increased with I-BET151 concentration in both cell lines: In total, 11% TSSs (3531) in the I-BET-sensitive MV4;11 and 1.6% TSSs (519) in the I-BET-resistant K562 showed significant displacement of BRD4 upon treatment with I-BET151 (Fig. 3a, b Additional file 6, 7, 8: Tables S3, S4 and S5) (“Materials and methods” section). Additionally, BRD4 displacement from the core promoter was more than from the pause site (Fig. 3b), in contrast to the higher BRD4 signal observed at pause site (Fig. 2a). Moreover, a considerable number of TSSs showed displacement at both the core promoter and pause site which was higher in MV4;11 than in K562 (Fig. 3b).

Notably, five TSSs in the sensitive MV4;11 cells showed significant BRD4 displacement upon treatment with 50 nM of I-BET151, while no significant binding loss was observed for the resistant K562 under the same condition (Fig. 3b). The five affected TSSs correspond to four non-coding RNAs: *LINC01585*, *LINC*-*ROR*, *NRON*, *LINC02367* and one protein coding gene, *RBM38*.

To further examine the I-BET151-dependent displacement of BRD4 upstream and downstream of the TSS, we used k-means clustering and split TSSs into four groups based on their ChIP-seq log fold change signal, independently for both cell lines (Fig. 3c and Additional file 9: Table S6). For MV4;11, clusters 1 and 2 (1274 and 450 TSSs, respectively) were characterized by medium and strong BRD4 displacement at + 1 Kb and − 1 Kb from the TSS and were named “sensitive core promoter and pause site” and “highly sensitive core promoter and pause site,” respectively. Clusters 3 and 4 (735 and 1072 TSSs, respectively) showed strong BRD4 binding loss at + 1 Kb or − 1 Kb from the TSS and were named “highly sensitive pause site” and “highly sensitive core promoter,” respectively (Fig. 3c; Additional file 10: Figure S4; Additional file 7, 8: Tables S4 and S5; “Materials and methods” section). Intriguingly, the four non-coding RNAs in which BRD4 shows a DB at 50 nM I-BET treatment (Fig. 3b) clustered in the highly sensitive promoter and pause site group (Fig. 3c,d cluster 2).

The same clustering approach was applied on the set of 519 TSSs in K562 (Fig. 3c, left panel). Interestingly, a similar clustering profile as for MV4;11 was identified in K562 with a balanced distribution of TSS in the four clusters (cluster 1:168 TSSs, cluster 2:108 TSSs, cluster 3: 132 TSSs and cluster 4: 111 TSSs).

By comparing the number of genes common to equivalent clusters from both cell lines, we found that only 55 genes belonging to the sensitive core promoter and pause site clusters were common in MV4;11 and K562, 31 genes belonging to the highly sensitive core promoter and pause site clusters, 19 genes belonging to the highly sensitive pause site clusters and 43 genes belonging to the highly sensitive core promoter clusters. This finding highlights differential sensitivity of promoters to I-BET-related displacement between the two cell lines.

To check whether differential BRD4 displacement is reflected on transcription, we quantified the expression of the five genes which showed BRD4 displacement exclusively in the I-BET-sensitive cells after 50 nM I-BET treatment. All five genes were downregulated in MV4;11 cells and either not detected or less affected in K562 (Fig. 3e). In MV4;11 cells, *LINC*-*ROR* showed a decrease in its expression, although not statistically significant (*p* value of 0.065; paired t-test). Since most long non-coding RNAs including *LINC*-*ROR* are known to have very low expression levels at steady state [45–48], the observed decrease in expression could have profound effects on its downstream targets.

The finding that four long non-coding RNAs with BRD4 binding at their promoters are among the most sensitive transcripts to I-BET in MV4;11 but not in K562 suggests a previously uncharacterized role for non-coding RNAs in conferring sensitivity to I-BET treatment.

### Chem-seq is predictive of differential effects of I-BET151 at promoters

**Fig. 4:**
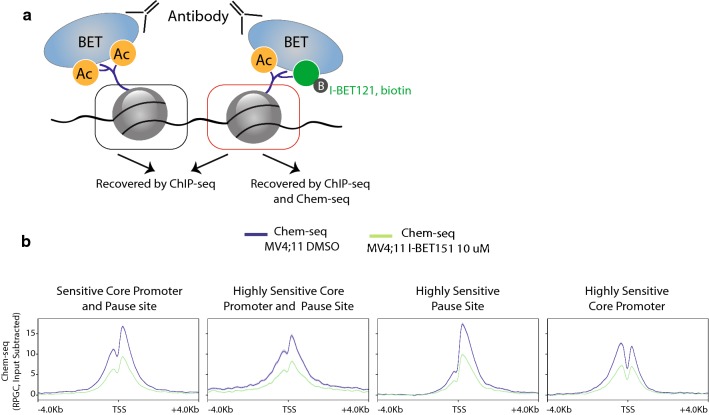
Chem-seq shows cluster-dependent signal profiles of BRD4/I-BET151 interactions. **a** Method scheme featuring loci recovered by ChIP-seq alone and those recovered by ChIP-seq and Chem-seq. Using an antibody against a BET protein member, we can recover all genomic loci bound by the protein. Using a biotinylated derivative of I-BET121, we can pull down fragments where the BET protein exhibits an accessible bromodomain. **b** Normalized genome-wide cluster-specific Chem-seq profiles on TSS −/+ 4 Kb in the absence or presence of excess free I-BET151 as control

In order to map genome-wide BRD4/I-BET151 interactions, we used a chemical affinity capture-based method, Chem-seq [11, 24] (Fig. 4a, “Materials and methods” section). This approach can both define loci where the BET protein BDs are accessible to the inhibitor and potentially actionable, thereby identifying different BET protein populations on chromatin. For this, we used a biotinylated derivative of I-BET121 (pan-BET inhibitor chemically similar to JQ1) [15] to pull down the associated chromatin fragments in cross-linked I-BET-sensitive MV4;11 cells followed by high-throughput sequencing. To control for specificity of binding, the pull down was also performed after addition of excess free I-BET151 (Fig. 4b). Interestingly, Chem-seq showed stronger signal enrichment downstream of the TSS for genes belonging to both the sensitive and highly sensitive core promoter and pause site clusters as well as the highly sensitive pause site cluster (clusters 1, 2 and 3) (Figs. 3c, 4b). In particular, the highly sensitive pause site cluster genes characterized by BRD4 DB only downstream of the TSS showed the highest signal in Chem-seq.

Conversely, TSSs belonging to the highly sensitive core promoter cluster (cluster 4) showed a typical bimodal distribution with no notable enrichment downstream or upstream of the TSS after Chem-seq. Chemical affinity capture of BET proteins bound to chromatin occurs mainly via the BD2 domain and thus is a measure of BD2 occupancy [24]. This observation indicates that BET proteins strongly displaced by I-BET treatment downstream of the TSS are characterized by greater availability of the BD2 domain. Stronger Chem-seq profiles downstream of the TSS indicate higher accessibility to I-BET treatment and might be used to predict gene responsiveness to BET protein inhibition.

### I-BET treatment induces apoptotic-related pathway signatures in the sensitive cell line

Next, we were interested to know whether I-BET treatment also affected global gene expression differentially in resistant and sensitive cell lines and if so, whether the differentially affected genes are connected to specific cellular pathways.

**Fig. 5:**
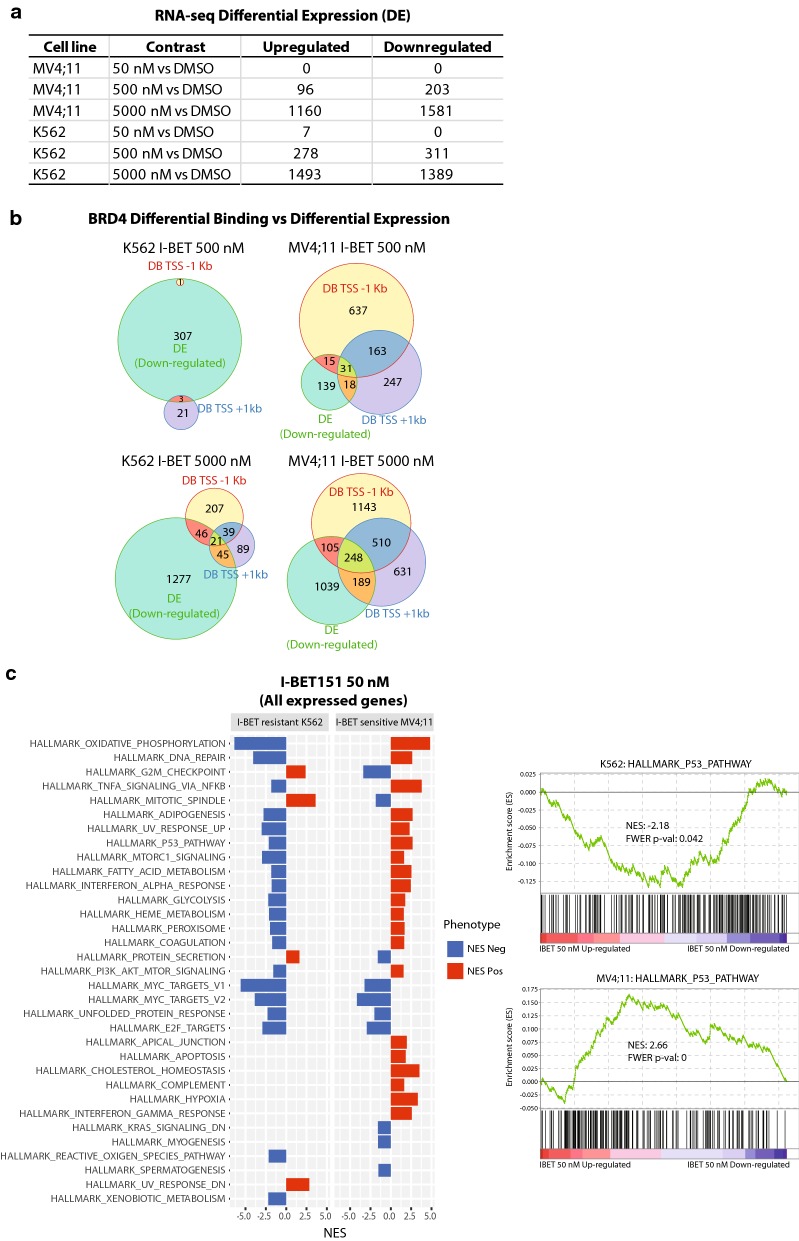
Gene set enrichment analysis (GSEA) defines hallmarks of I-BET sensitivity. **a** Table summarizing counts of differentially expressed genes for all conditions in both cell lines. **b** Venn diagrams representing the counts of common or distinct sets between genes exhibiting BRD4 differential binding (DB) and genes exhibiting differential expression (DE) for each cell line and I-BET concentration. Counts and overlaps for the I-BET151 50 nM treatment were too small and thus not represented here. **c** Normalized enrichment scores (NES) of GSEA for hallmark gene sets v6.1. Listed are all hallmarks that show significant NES (− 1 < NES or NES > 1 with FDR < 0.1) in MV4;11 or K562 treated with I-BET 50 nM compared to DMSO. Log2 fold changes of gene expression were used to identify enriched sets. Red bars correspond to enriched gene sets for upregulated genes and blue for downregulated genes. The enrichment profiles for “Hallmark P53 Pathway” for both cell lines are shown on the right as an example

RNA-seq was performed in MV4;11 and K562 cells treated with 50, 500 or 5000 nM I-BET151 (“Materials and methods” section), and differentially expressed genes were called using DESeq2 [44]. As expected, the number of deregulated genes increased with increasing I-BET concentrations in both cell lines (Fig. 5a) (Additional file 11, 12: Tables S7 and S8).

To correlate BRD4 displacement from promoter regions with corresponding gene expression at a global level, we compared the number of genes showing differential BRD4 binding at TSS to those exhibiting differential expression. Although both MV4;11 and K562 exhibited a similar number of downregulated genes (Fig. 5a), the degree of overlap between differentially bound and downregulated genes was higher for the sensitive MV4;11 cell line (Fig. 5b), which suggests a higher number of direct effects of the I-BET drug in the sensitive MV4;11 cell line. For instance, at I-BET 5000 nM, 34.3% (542/1581) of downregulated genes in MV4;11 exhibit differential binding within 1 Kb upstream and downstream of the TSS compared to only 8% (112/1389) in K562.

We then identified enriched pathways using gene set enrichment analysis (GSEA) [49] in pre-ranked mode using all genes following I-BET treatment (“Materials and methods” section) in both cell lines. Interestingly, several genes in pathways linked to cell viability were differentially affected at 50 nM I-BET treatment in both cell lines (Fig. 5c, Additional file 13: Figure S5). For instance, genes in the P53 pathway were upregulated in the I-BET-sensitive MV4;11 (normalized enrichment score or NES = 2.6, FDR FWER *p* value = 0) and conversely downregulated in the I-BET-resistant K562-treated cells (NES = − 2.13, FWER *p* value = 0.056). Although K562 cells do not express a functional form of TP53, the P53 pathway is known to be functional in this cell line [50]. Similar differences between the two cell lines were observed for TNF-alpha signaling via NF-kappaB, DNA repair and apoptosis pathways (Fig. 5c). Our results suggest that MV4;11 sensitivity to I-BET treatment is manifested at the transcriptional level by an upregulation of gene sets belonging to pathways which drive cells into cell cycle arrest and/or cell death and oxidative phosphorylation and that an opposite response is shown in the resistant cell line K562. At higher inhibitor concentrations, more pathways become similarly affected in both cell lines (Additional file 13: Figure S5), most likely because the resistant K562 cell line also becomes more responsive to I-BET.

### BRD4 displacement downstream of the TSS prolongs RNA Pol II pausing and decreases gene expression

**Fig. 6:**
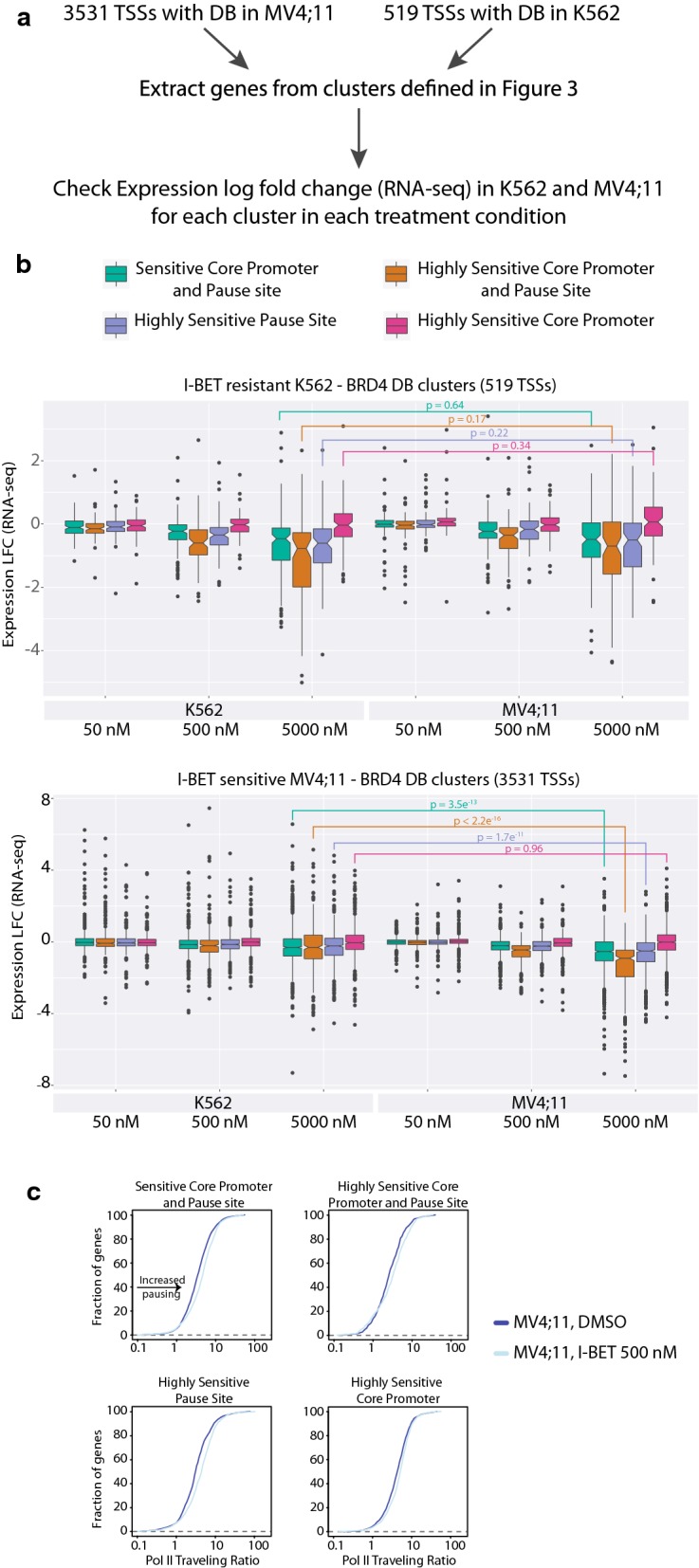
Gene expression changes correlate with a significant loss of BRD4 binding at − 1 Kb and with increase in Pol II pausing. **a** Schematic representation of the analysis performed to correlate the signature of DB with DE. **b** Boxplots for RNA-seq log2 fold changes for each cluster identified in Fig. 3c as a function of cell type and condition: upper panel for K562-based clustering and lower panel for MV4;11-based clustering. *P* values correspond to Wilcoxon signed-rank test. **c** Traveling ratio (TR) based on Pol II ChIP-seq in MV4;11 cells: Each panel represents TR of genes belonging to clusters defined in Fig. 3c in DMSO condition (blue) and in response to 500 nM I-BET treatment (light blue)

BRD4 displacement at promoters has been linked to downregulation of its target genes. Since BRD4 is essential for transcription elongation which starts downstream of the TSS, we postulated that BRD4 displacement at 1 Kb downstream of the TSS, spanning the Pol II pause site, is mostly responsible for affecting gene expression. To test for this hypothesis, we compared gene expression of the different DB clusters identified above (Fig. 3c) between K562 and MV4;11 (Fig. 6a).

Genes with sensitive and highly sensitive core promoter and pause site to I-BET in MV4;11 cells (i.e., belonging to clusters 1 and 2) are highly downregulated by I-BET in this cell line but not in the I-BET-resistant K562 cells (Fig. 6b, Wilcoxon test *p* = 3.5e^−13^ and *p* < 2.2 e^−16^, respectively). The expression of genes belonging to clusters 1 and 2 defined in K562 is similarly affected by I-BET treatment in both cell lines (Fig. 6b, Wilcoxon test *p* = 0.64 and *p* = 0.17, respectively). Most interestingly, genes belonging to the highly sensitive pause site cluster in MV4;11 showed a very significant reduction in expression in this cell line compared to the corresponding category of genes in K562 (Fig. 6b, Wilcoxon test *p* = 1.7e^−11^). Moreover, this is in strong contrast to the genes in the highly sensitive core promoter cluster that show no reduction in expression in the sensitive cell line MV4;11 (*p* = 0.96).

Since BRD4 is important for both transcription initiation and elongation, these results suggest that the strongest loss of expression observed using I-BET151 in the sensitive MV4;11 cell line is due to BRD4 displacement at the Pol II pause site, downstream of the TSS, which likely impairs transcription elongation [51]. This is in agreement with our Chem-seq data (Fig. 4b), where sensitive and highly sensitive core promoter and pause site clusters, as well as the highly sensitive pause site cluster, are most accessible to the drug. In contrast, genes belonging to the highly sensitive core promoter cluster (cluster 4) showed no topological signal bias by Chem-seq and their expression was not affected by I-BET.

If BRD4 displacement in the highly sensitive pause site cluster impairs transcription elongation, we would expect to observe an accumulation of Pol II binding at the promoter region, as opposed to the gene body, following I-BET treatment. This will affect the pause state of many genes, thus leading to an increase in their pause index (PI), also known as travelling ratio (TR). To test this hypothesis, we compared the TR for all four MV4;11 clusters before and after treatment with 500 nM I-BET [52] (“Materials and methods” section). Pol II traveling ratio was affected in all four clusters following I-BET treatment of the sensitive cell line MV4;11 (Fig. 6c). Interestingly, the pause site sensitive cluster showed a more pronounced increase in Pol II TR when compared to other clusters after drug treatment. In fact, 7.3% genes (55/732) gained in TR (TR > 2) in the highly sensitive pause site cluster compared to 3.6% (38/906) in the highly sensitive core promoter cluster, upon I-BET treatment (*p* value of 0.00031; two-sided proportion test). For the sensitive and highly sensitive core promoter and pause site clusters, 5.4% (68/1265) and 6.3% (28/444) genes displayed an increase in their TR, respectively, when compared to the core promoter sensitive cluster (*p* values of 0.046 and 0.025, two-sided proportion test) (Fig. 6c).

To summarize, sensitivity of MV4;11 cells to I-BET is characterized by a strong displacement of BRD4 downstream of the TSS which coincides with a longer pausing of RNA Pol II and a subsequent decrease in gene expression.

## Discussion

Quantitative analysis of drug response enables the evaluation of compound-effect relationship and allows us to distinguish specific and direct outcomes of compound treatment from random and downstream consequences. Epigenetic drugs function by engaging their target(s) on chromatin, which usually induces or modifies a transcriptional response. Albeit the consequences of I-BET binding to their targets have been extensively studied [10], a genome-wide quantitative analysis of biochemical and functional sensitivity to I-BET and its causal relationship to intrinsic cellular drug resistance, has not been explored in detail. By using two leukemia cell lines which show intrinsic sensitivity or resistance to I-BET, we performed biochemical and functional studies after treating these cells with increasing concentrations of I-BET151. We compared the I-BET concentration-dependent chromatin displacement of all three BET paralogs in both sensitive and resistant cell lines. In particular, we found that the decrease in BRD4 ChIP signal at promoters, typical and super-enhancers is clearly more prominent in the I-BET-sensitive MV4;11 than in the I-BET-resistant K562 cells at the lowest I-BET151 concentration used (Fig. 2 and Additional file 5: Figure S3). This is not the case for BRD2 and BRD3 which show similar displacement levels at 50 nM I-BET treatment in both cell lines (Fig. 2 and Additional file 5: Figure S3). These data agree with the higher sensitivity of the AML cell line (MV4;11) to I-BET and suggest that BRD4 is the major target governing I-BET sensitivity. Although I-BET151 has a similar affinity for each of the BET proteins [15], the fact that BRD4 has a unique role in assembling a processive RNA Pol II complex [13, 14] might render this BET paralog more accessible to the inhibitor at promoter proximal sites.

Gene set enrichment analysis revealed that the expression of genes belonging to proliferation/apoptosis-associated pathways is differentially regulated between the two cell lines upon I-BET treatment. Upregulated genes of the P53 pathway were mostly enriched in the I-BET-sensitive MV4;11 cells and downregulated in K562 cells (Fig. 5c). Similarly, genes in the oxidative phosphorylation pathway were upregulated in MV4;11 and downregulated in K562 cells. Conversely, genes belonging to the G2 M checkpoint and mitotic spindle pathways were mostly downregulated in MV4;11 but upregulated in K562. Taken together, our results indicate that metabolic activities as well as signaling pathways characteristic of proliferating cancer cells (reviewed in [53]) are mostly impaired in MV4;11 compared to K562 as a consequence of BET protein inhibition.

Loss of BET protein binding at enhancers and at TSSs is a direct explanation for the I-BET-induced changes in transcription. In I-BET-sensitive MV4;11 cells, five genes, in particular, showed a prominent loss of BRD4 signal at the lowest I-BET concentration (Fig. 3b). Interestingly, four of these genes are lncRNAs and one is an RNA binding protein which is involved in AML cell differentiation [54]. This is the first study, to our knowledge, showing the sensitivity of lncRNAs to I-BET treatment and warrants further mechanistic studies in the area.

The long intergenic non-coding RNA *LINC*-*ROR* is overexpressed in several human cancers and has been implicated in tumorigenesis in particular because of its stabilizing function of c-Myc RNA [55], a hallmark of I-BET action, and suppression of the p53 pathway [56] which is clearly upregulated in the I-BET-sensitive MV4;11 upon I-BET treatment (Fig. 5c).

Pol II binding at TSSs and release after stalling mark transcription initiation and elongation phases in which BRD4 plays an essential role [57]. Although several thousand TSSs are occupied by BET proteins, and I-BETs are known to displace their targets from chromatin, not all BET protein bound genes show the same change in expression following I-BET treatment. A spatial resolution analysis of BRD2, BRD3 and BRD4 ChIP data has revealed that different patterns of BET protein binding and I-BET-driven displacement exist at TSSs which differentially alters the expression of downstream genes. All three BET paralogs display a bimodal binding pattern at most TSSs characterized by two peaks of ChIP signal, at approximately 150 bp upstream and 300 bp downstream from the TSS which resemble the initiating and stalling of Pol II, respectively [58]. The relative intensities of BET protein signals at both peaks before and after compound treatment drove the clustering of TSSs into four distinct biochemical categories. Correlation of these biochemical categories with transcriptomic data, translated these clusters into defined I-BET sensitivity gene groups. Strong binding and displacement at 1 Kb downstream of the TSS, encompassing the Pol II pause site, defined the genes whose transcription was mostly affected by I-BET. Pol II release from stalling occurs at sites downstream of the TSS [59] and is mediated by pTEFb phosphorylation of Pol II at Ser2. Since pTEFb is known to be recruited to chromatin by BRD4, displacement of the population of BRD4 downstream of the TSS by I-BET would also remove pTEFb and thus impair Pol II release from stalling. Indeed, Pol II pausing was mostly affected for the genes characterized by a stronger displacement of BRD4 downstream of the TSS (Fig. 6c) in line with two recent studies [51, 60]. Conversely, the displacement of the BRD4 population at the core promoter alone (defined as 1 Kb upstream of the TSS) was not enough to induce a transcriptional response unless combined with BRD4 displacement downstream of the TSS; this pattern resulted in a pronounced gene downregulation (Fig. 6b). BET proteins bound upstream of the TSS might constitute a deposit which is used to replenish the transcriptionally critical BET protein population downstream of the TSS. An intriguing hypothesis is that BRD4 upstream of the TSS contributes to the recruitment of transcription factors: The fact that BRD4 bound upstream of the TSS is not efficiently recovered by chemical pull downs (Fig. 4b), might indicate that its BDs, in particular the BD2, are not accessible for binding or occupied [24]. The BD2 domain can bind transcription factors which mediate the docking of Pol II at initiation sites [61]. Interestingly, several general transcription factors (GTFs) have been found to co-purify with BRD4 [15]. Displacement of this BET protein pool bound upstream of the TSS might impair further transfer of BRD4 to Pol II pausing–release sites downstream of the TSS and result in enhanced or more durable inhibition of Pol II release. This would explain why strong displacement at both TSS upstream and downstream BRD4 binding sites (highly sensitive core promoter and pause site cluster) results in very efficient gene downregulation.

The four biochemical categories of TSSs as defined by location of BET proteins binding and fold displacement upon I-BET treatment, are present in both I-BET-resistant K562 cells and I-BET-sensitive MV4;11 cells. Moreover, the relationship between a given biochemical TSS category and effect in gene expression is conserved between the two model cell lines. What distinguishes the I-BET-sensitive from the I-BET-resistant leukemia cells is the stronger decrease in gene expression of commonly affected TSSs (Fig. 6b) and the presence of a set of genes which is solely affected in MV4;11 (Fig. 3).

## Conclusions

Several studies have brought evidence for pathways and mechanisms to explain the acquired sensitivity of cancer cells to I-BET proteins [41, 60, 62]. Yet, no unifying hypothesis which would enable mechanistic prediction for sensitivity to BET proteins inhibition can be derived. Our genome-wide comparative analysis of chromatin-bound BRD2, BRD3 and BRD4 to I-BET dosing identified a cell and gene-specific BRD4 response which can be correlated with drug sensitivity. Moreover, a high-resolution spatial analysis of BRD4 signals at TSSs revealed a bimodal signature of BRD4 binding and I-BET-mediated BRD4 displacement which affects Pol II pausing and, as a consequence, gene expression of specific gene sets. Altogether, this approach provides a functional and mechanistic explanation of why certain leukemia cell lines are more sensitive than other to BET proteins inhibition and defines guidelines for an unbiased prediction of intrinsic cellular sensitivity to I-BET treatment.

## Materials and methods

### Cell proliferation assays

MV4;11 and K562 cells were grown in RPMI-1640 supplemented with 10% FBS, in a humidified incubator (37 °C, 5% CO2). Cells were seeded at 5000 cells/well in 96-well plates, 1 µl of I-BET151 was added per well from 100 × DMSO stocks (final DMSO concentration: 1%), and cells were incubated for 72 h. Cell viability was measured using CellTiter Glo assay kit (Promega) following the manufacturer’s instructions. The response area in Fig. 1a, which corresponds to 1-viability, was calculated using the “computeAUC” function from the PharmacoGX package in R [63].

### Chromatin immunoprecipitation (ChIP) and Chem-seq

ChIP-seq and Chem-seq assays were performed as described previously [24]. Briefly, K562 and MV4;11 cells were treated with either DMSO or increasing concentration of I-BET151 for 6 h at 37 °C. After treatment, cells were cross-linked with 1% formaldehyde for 10 min at room temperature, and cross-linking was stopped by the addition of 0.125 M glycine. Cells were then lysed using the SimpleChIP^®^ Enzymatic Chromatin IP Kit (CST #9004), and DNA was digested to mostly mononucleosomes as recommended by the manufacturer.

For ChIP-seq, anti-BRD4 (Bethyl labs #A301-985A), anti-BRD3 (Bethyl Labs #A302-368A) and anti-BRD2 (Bethyl Labs #A302-583A) antibodies were added to the lysate and incubated for 3 h at 4 °C. Protein G magnetic beads (Thermo Scientific #10004D) were then added and incubated for 1 h at 4 °C. The lysate was then aspirated, and the beads were washed twice with 200 µl SimpleChIP^®^ Chromatin IP Buffer (#14231), once with SimpleChIP^®^ Chromatin IP Buffer containing 350 mM NaCl, once with SimpleChIP^®^ Chromatin IP Buffer containing 125 mM LiCl and once with Tris–EDTA buffer. Beads were eluted with SimpleChIP^®^ elution buffer by incubation at 65 °C for 30 min. After addition of 200 mM final NaCl, samples were placed at 65 °C overnight for reverse cross-linking. ChIP conditions were optimized for each protein by ChIP-qPCR using the recovery of positive and negative regions. For this, the positive primer set used to target the ZC3H4 locus was AATGCACGAAAAAGGACTGC as forward and TATACCCCATTCCCATCAGG as reverse. The negative primers were ordered form Active Motif ^®^ (http://www.activemotif.com/catalog/764/human-control-qpcr-primer-sets).

For Chem-seq, lysates from untreated cells were incubated either with DMSO or with 10μM I-BET121 for 2.5 h at 4 °C. Dynabeads MyOne Streptavidin T1 (Thermo Scientific #65602) coupled to biotinylated I-BET121, were added to the lysate and incubated for 1 h at 4 °C. After washing, beads were eluted by incubation in elution buffer at 65 °C for 30 min. After addition of 200 mM final NaCl, samples were placed at 65 °C overnight for reverse X-linking.

### ChIP and RNA library construction

Library preparation of immunoprecipitated DNA was done using the NEBNext^®^ ChIP-Seq Library Prep Master Mix Set for Illumina^®^ (NEB), and samples were sequenced in a 50 bp single end run on the Illumina HiSeq-2000.

Barcoded stranded mRNA-seq libraries were prepared from high-quality total RNA samples (~ 200 ng/sample) using the Illumina TruSeq RNA Sample Preparation Kit (Illumina, San Diego, CA, USA) implemented on the liquid handling robot Beckman FXP2. Obtained libraries that passed the QC step were pooled in equimolar amounts; 10 pM solution of this pool was loaded on the Illumina sequencer HiSeq 2000 and sequenced unidirectionally, generating ~ 180 million reads (30 million reads per each library), each 50 bases long.

### RT-qPCR

MV4;11 and K562 cells were cultured as described above, plated in 6-well plates and treated for 6 h with either DMSO or I-BET151 (50 nM, final DMSO concentration of 0.02%). Four replicates were cultured per condition. Cells were lysed immediately after collection, and lysates were stored at − 80 °C. RNA was extracted using the Rneasy mini kit (Qiagen, #74104). cDNA was synthesized using the Protoscript 2 kit (NEB, #E6560S) according to manufacturer’s specifications. qPCR was performed in duplicate using 0.9 µl cDNA per reaction, default SYBR green cycling settings (StepOne software), StepOnePlus thermocyclers (Thermo Fischer, #4376600) and SYBR Green PCR master mix (Thermo Fischer, # 4309155).

Reference transcripts were chosen based on RNA-seq data with the following criteria: (1) unaffected by the experimental treatment (nonsignificant expression changes up to 500 nM I-BET 151 treatment) and (2) expression higher than 0.8 quantile in both K562 and MV4;11.

Out of three reference genes having the requirements listed above (*otud5*, *nap1l1* and *tm9sf4*), otud5 was chosen as reference since it exhibited the smallest change in expression between DMSO and I-BET151-treated samples in both cell lines.

qPCR data were analyzed using the following formula which corresponds to the adaptation of the Delta Ct method, considering individual primers’ efficiency with “X” representing the target transcript and “R” the reference transcript.

$$X = \frac{{(1 + EffR)^{CtR}}}{{(1 + EffX)^{CtX}}}$$

The primers used are: *RBM38* (CACCTTGATCCAGCGGACTTA, GGTGGGTAGATGTAGTGCGG), *LOC101930452* (CCCAATTCACAGCCGCATTT, TATCGGGGGCAGCTCTTACT), *LINC01585* (ACTCTTAAAGCCACCAGCCC, CTGCATTTCACCGAAGGTCG), LINC-*ROR* (ACTCCAGCTATGCAGACCAC, ACCTTTCCACACACCTGTCC), *NRON* (CATGGCGACGGCAAAATCAT, AGCTGCCGGCATGATAAGAA), *OTUD5* (CCAGTACAGCACAGAACCCA, GGGTTCGTCCTCGTTTTGATG).

### Super-enhancers analysis

A list of MV4;11 enhancers and super-enhancers were obtained from the Dawson lab [24]. They consist of 14,442 (median size of 27,481 bp) and 327 (median size = 1046 bp) enhancers and super-enhancers, respectively. K562 enhancers and super-enhancers were downloaded from dbSUPER [64]. For K562, the data consist of 11,281 enhancers (median size = 875 bp) and 742 super-enhancers (median size = 17 602 bp). To generate binding profiles over enhancers and super-enhancers, intergenic enhancers were considered and defined as enhancers with no overlap with a TSS −/+ 1 Kb. In this case, K562 has 9333 enhancers (median size = 1302 bp) and 200 super-enhancers (median size = 19,004 bp). For MV4;11, we defined 11,416 and 226 enhancers and super-enhancers, respectively, not overlapping a TSS −/+ 1 Kb (median sizes = 25,782 bp and 895 bp, respectively).

### ChIP-seq and Chem-seq peak calling

To overcome limitations in peak calling algorithms for factors with broad signal, including chromatin readers, we derived a macs2 [38]-based pipeline which takes advantage of the two replicates for each condition. For each condition, we merged the two replicates to create one single bam file. We then split the merged file into two pseudo-replicates containing equal number of reads (Additional file 2: Figure S1) and used macs2 to call peaks on the merged set and the two pseudo-replicates independently, using input as a control (call peak -t target -c control -f BAM -n name –outdir outdir –g 2.7e9 -p 1e^−3^ –broad –broad-cutoff 1e^−3^). We then obtained the pre-final set of peaks by considering common peaks to the tree runs performed above. Peaks were then filtered for ENCODE blacklisted regions corresponding to regions with anomalous and high signal/read counts using bedtools [65] leading to the final set of peaks.

#### ChIP-seq and Chem-seq analysis

Quality check for raw sequencing reads was performed using FastQC [66] mainly to monitor duplication rates and sequence contamination. Reads were aligned using BWA [67] with default parameters and hg19 as reference genome “bwa aln -t 10 hg19 in.fastq > out.sai” and “bwa samse hg19 in.sai > out.sam”. Samtools [68] was used for SAM to BAM conversion and duplicate removal. Normalized Bigwig profiles for Chip-seq and Chem-seq were obtained using bamCoverage from deeptools [69] by using a binSize of 50, a read extension of 300 and the “normlaizeTo1x” as normalization option. For input subtraction, we used bamCompare from deeptools and “–ratio subtract” as option for subtraction. Profiles around TSSs were plotted using the computeMatrix and plotProfile from deeptools [69] from bigwig profiles normalized using the RPGC method with input subtracted. IGV [70] was used for the visualization of bigwig profiles, manual inspection and generation of browser screenshots. Heatmaps (i.e., Fig. 3) were generated using the R package ComplexHeatmap [71]. K-means clustering was applied with K = 4 using the “ward.D” as clustering method.

Pol II Traveling ratio was calculated by dividing read density on promoter, defined as − 100 bp to + 300 bp from TSS, by read density on gene body defined as 301 bp from TSS until end of gene.

MV4;11 raw data and peaks (fastq files) for H3K27ac, H4K5ac and Pol II were downloaded from NCBI GEO (GSE71780) [52]. Analysis was carried the same way as ChIP-seq datasets generated for this study mainly for generation of bigwig profiles for visualization using deeptools [69]. K562 H3K27ac peaks were obtained from ENCODE project database (GEO:GSM733656) [72].

### Differential binding analysis

RefSeq TSSs were downloaded from UCSC genome browser (hg19), and duplicate records were removed. Two categories were created using bedtools slop [65] in order to create TSS − 1 Kb and TSS + 1 Kb records. Read summarization was performed using featureCounts [73] followed by differential binding analysis using DESeq2 [44].

### RNA-Seq analysis

Reads were aligned to the human genome (G1k V37) with Tophat2 [74] and Bowtie2 [75], and reads were assigned to genes with htseq-count [76]. Differential expression analysis was performed with DESeq2 [44] in the R statistical programming language. Genes with a false discovery rate (corrected for multiple testing with the method of Benjamini and Hochberg) below 0.05 and an absolute log2 fold change greater than 1 were considered to be significantly differentially expressed. Conversion between name ID, mainly between symbols and Refseq or Ensembl IDs, was done using R. Gene set enrichment analysis (GSEA) was run using pre-ranked mode for all genes based on log2 fold change derived from the differential expression analysis. Pathways with a normalized enrichment score > 1 or < − 1 at FDR < 10% were considered significant.

## Additional files

**Additional file 1: Table S1.** Counts of raw and processed reads for each assay (ChIP-seq or Chem-Seq), protein (BRD2/3/4), condition (DMSO, 50 nM, 500 nM or 5000 nM) and cell line (MV4;11 and K562) used. Percentage of uniquely mapped reads is also shown.

**Additional file 2: Figure S1.** Diagram depicting the strategy used to call peaks described in Materials and Methods.

**Additional file 3: Figure S2.** Barplot with the number of peaks called for each condition and for both cell lines (a) for all peaks and (b) for peaks overlapping a TSS − 1 Kb or a TSS + 1 Kb. (c) Stacked barplot showing the percentage of peaks falling in TSS −/+ 1 Kb (blue), H3K27ac marked regions excluding TSS −/+ 1 Kb (orange) and other intergenic or genic regions excluding TSS −/+ 1 Kb and H3K27ac marked sites (gray) after treating cells in DMSO, 50 nM, 500 nM or 5000 nM I-BET151.

**Additional file 4: Table S2.** Number of peaks called for each condition and cell line, peaks falling on the TSS −/+ 1 Kb, those present in H3K27ac marked regions excluding TSS −/+ 1 Kb and remaining peaks.

**Additional file 5: Figure S3.** Genome-wide ChIP-seq profiles on intergenic typical enhancers and super-enhancers for BRD2, BRD3 and BRD4 after treating cells in DMSO and with different concentrations of I-BET151. Upper panel shows the profiles for K562 and lower panel for MV4;11. All ChIP-seq profiles are RPGC (Reads Per Genomic Content) normalized with input subtraction. Plots are generated with the same y-scale for comparison purposes. “Start” and “End” for typical and super-enhancers were based on the median size of the corresponding enhancer category.

**Additional file 6: Table S3.** Number of events with differential binding at TSS − 1 Kb and + 1 Kb. In addition to information in Fig. 3b, this table shows the number of TSSs affected in each condition for the indicated interval and also, the TSSs not affected in any condition (“unaffected” TSS). Percentages of affected TSSs at − 1 Kb, + 1 Kb or both is also indicated.

**Additional file 7: Table S4.** Table of DESeq2 differential binding (DB) results for all TSSs in MV4;11. TSSs are represented by their gene name followed by the Refseq ID in the form name_REFSEQ_ID. This nomenclature allows us to differentiate between TSSs belonging to different transcripts of the same gene. Column names contain the information on the protein (BRD4) and the inhibitor concentration, i.e., mb4_50_1 Kb_Up.log2FoldChange corresponds to the log2 fold change of the DB analysis on MV4;11 (m), for the protein BRD4 (b4) at 50 nM of inhibitor (50) for TSS − 1 Kb (1 Kb_UP).

**Additional file 8: Table S5.** Same as supplementary Table 4 but for K562.

**Additional file 9: Table S6.** List of all clusters and TSSs belonging to each of them (represented by their gene and transcript name) in MV4;11 and K562 as defined in Fig. 3c.

**Additional file 10: Figure S4.** Genome browser visualization of a locus from each of the 4 clusters defined in MV4;11 in Fig. 3c. Genome browser showing the decrease in ChIP signal for all BET proteins when treated with I-BET151. Tracks are as in Fig. 2c: the dashed vertical line marks the position of the TSS and the green box marks the surrounding upstream and downstream 1 Kb regions from the TSS. The scale is shown in the lower left corner.

**Additional file 11: Table S7.** Table of DESeq2 differential expression (DE) in MV4;11 representing for each gene (ensID and symbol) the log fold change and adjusted p value for all inhibitor concentrations using DMSO as a reference.

**Additional file 12: Table S8.** Same as supplementary Table 7 but for K562.

**Additional file 13: Figure S5.** Gene set enrichment analysis for all I-BET treatment conditions in both cell lines. Normalized enrichment scores (NES) of GSEA for hallmark gene sets v6.1. Listed are all hallmark that shows significant NES (− 1 < NES or NES > 1). Log2 fold changes of gene expression in MV4;11 or K562 treated with I-BET 50 nM, 500 nM or 5000 nM compared to DMSO were used to identify enriched sets. Red bars correspond to enriched gene sets for upregulated genes and blue for downregulated genes.

## Data Availability

Raw sequencing data have been deposited to GEO under accession number GSE120715 along with processed bigwig and bed for ChIP-seq and Chem-seq and counting files for RNA-seq. The derivative of I-BET121 used for Chem-seq is available under MTA.

## Abbreviations

ChIP: chromatin immunoprecipitation
Chem-seq: chemical affinity capture method followed by sequencing
BET: bromo- and extra-terminal domain
TSS: transcription start site
DB: differential binding
DE: differential expression
BD: bromodomain
I-BET: inhibitor of BET protein
Kb: kilobase(s)
