# Supplementary figures and images for: BRD4 bimodal binding at promoters and drug-induced displacement at Pol II pause sites associates with I-BET sensitivity
Source: Epigenetics Chromatin. 2019 Jul 2;12:39. doi: 10.1186/s13072-019-0286-5 (PMC6604197; doi:10.1186/s13072-019-0286-5)

Supplementary Figure 1, Khoueiry, Gahlawat et al.

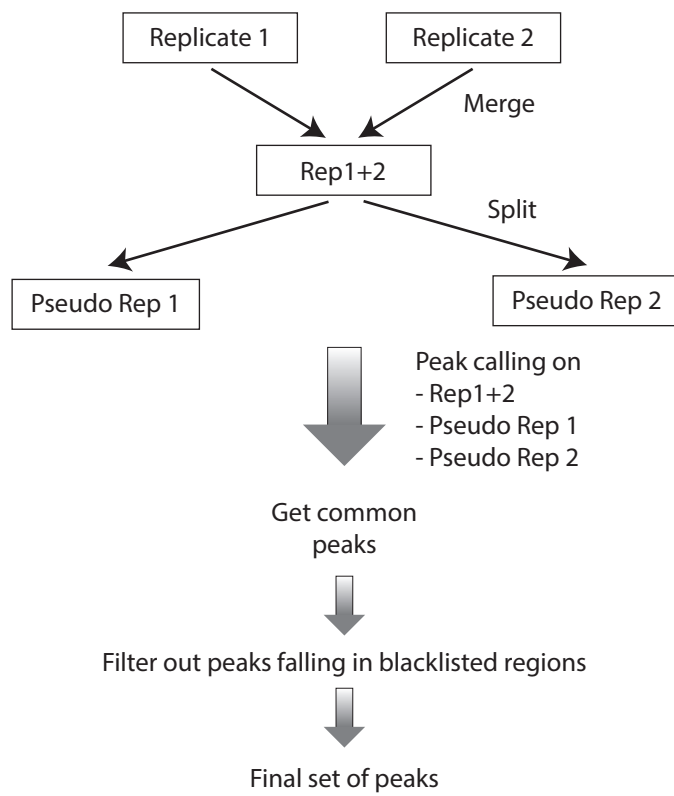

Supplement: Supplementary file 2 — Additional file 2: Figure S1. Diagram depicting the strategy used to call peaks described in Materials and Methods. [file 13072_2019_286_MOESM2_ESM.pdf]

a

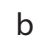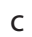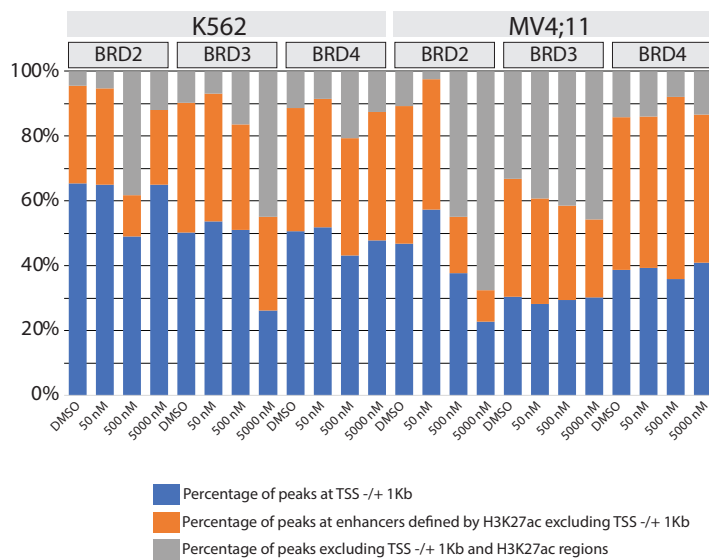

Supplement: Supplementary file 3 — Additional file 3: Figure S2. Barplot with the number of peaks called for each condition and for both cell lines (a) for all peaks and (b) for peaks overlapping a TSS − 1 Kb or a TSS + 1 Kb. (c) Stacked barplot showing the percentage of peaks falling in TSS −/+ 1 Kb (blue), H3K27ac marked regions excluding TSS −/+ 1 Kb (orange) and other intergenic or genic regions excluding TSS −/+ 1 Kb and H3K27ac marked sites (gray) after treating cells in DMSO, 50 nM, 500 nM or 5000 nM I-BET151. [file 13072_2019_286_MOESM3_ESM.pdf]

Supplementary Figure 3, Khoueiry, Gahlawat et al.

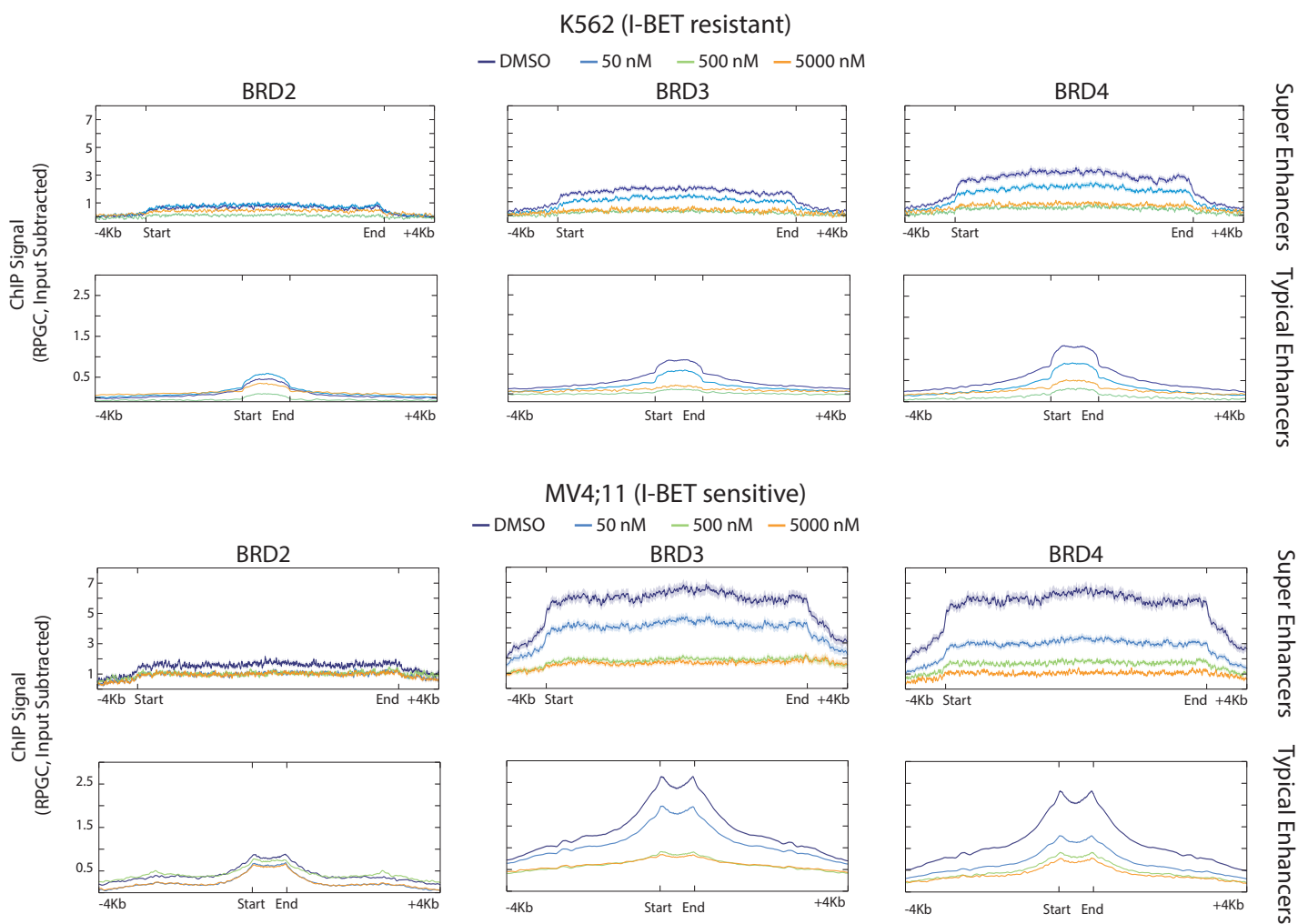

Supplement: Supplementary file 5 — Additional file 5: Figure S3. Genome-wide ChIP-seq profiles on intergenic typical enhancers and super-enhancers for BRD2, BRD3 and BRD4 after treating cells in DMSO and with different concentrations of I-BET151. Upper panel shows the profiles for K562 and lower panel for MV4;11. All ChIP-seq profiles are RPGC (Reads Per Genomic Content) normalized with input subtraction. Plots are generated with the same y-scale for comparison purposes. “Start” and “End” for typical and super-enhancers were based on the median size of the corresponding enhancer category. [file 13072_2019_286_MOESM5_ESM.pdf]

Supplementary Figure 4, Khoueiry, Gahlawat et al.

a

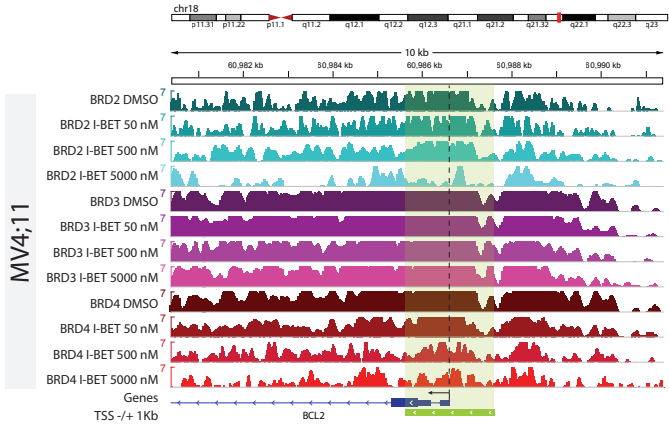

b

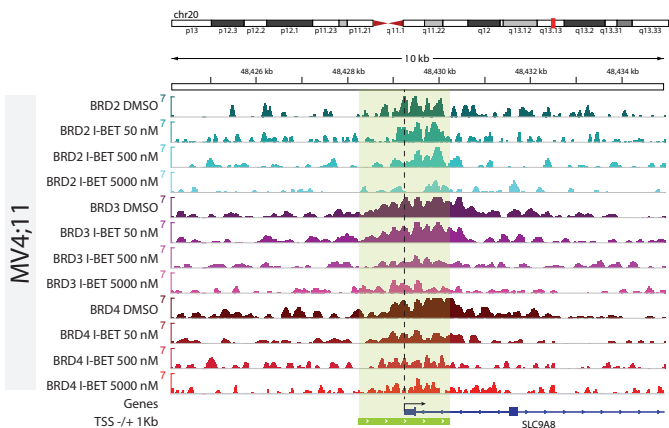

c

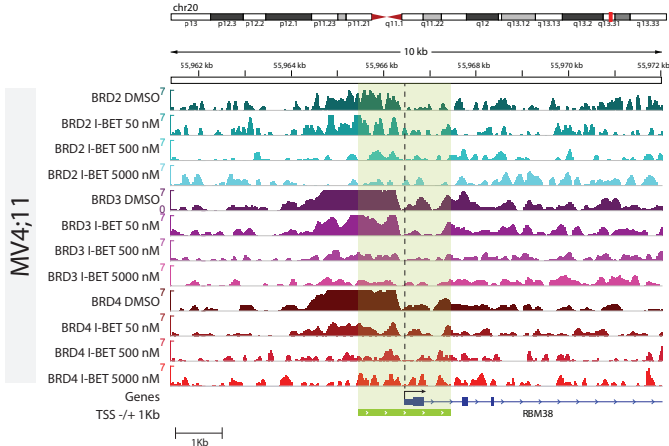

Supplement: Supplementary file 10 — Additional file 10: Figure S4. Genome browser visualization of a locus from each of the 4 clusters defined in MV4;11 in Fig. 3c. Genome browser showing the decrease in ChIP signal for all BET proteins when treated with I-BET151. Tracks are as in Fig. 2c: the dashed vertical line marks the position of the TSS and the green box marks the surrounding upstream and downstream 1 Kb regions from the TSS. The scale is shown in the lower left corner. [file 13072_2019_286_MOESM10_ESM.pdf]

Supplementary Figure 5, Khoueiry, Gahlawat et al.

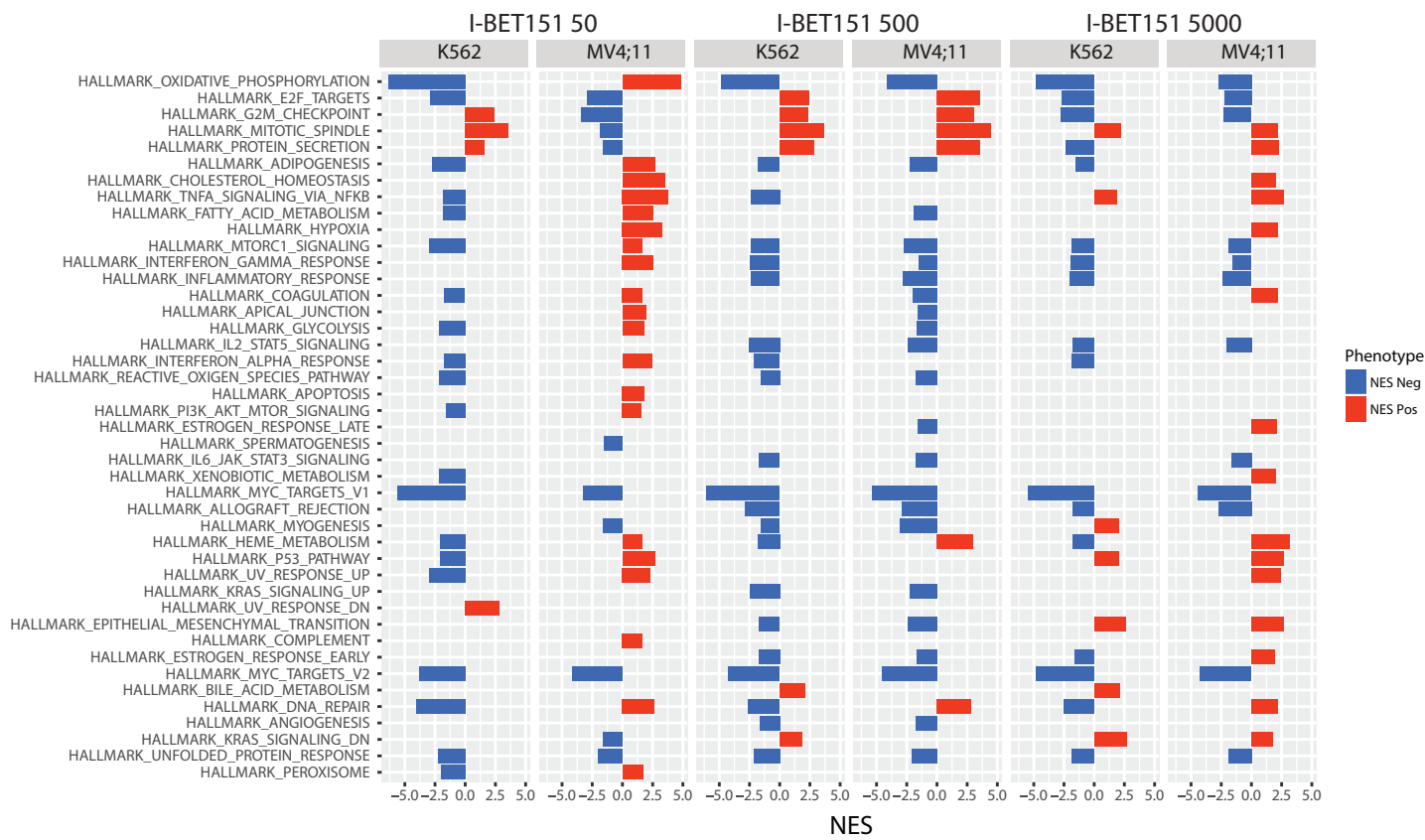

Supplement: Supplementary file 13 — Additional file 13: Figure S5. Gene set enrichment analysis for all I-BET treatment conditions in both cell lines. Normalized enrichment scores (NES) of GSEA for hallmark gene sets v6.1. Listed are all hallmark that shows significant NES (− 1 < NES or NES > 1). Log2 fold changes of gene expression in MV4;11 or K562 treated with I-BET 50 nM, 500 nM or 5000 nM compared to DMSO were used to identify enriched sets. Red bars correspond to enriched gene sets for upregulated genes and blue for downregulated genes. [file 13072_2019_286_MOESM13_ESM.pdf]
